# A conservation organisation’s approach to COVID-19: Lessons learned from Madagascar

**DOI:** 10.4102/jamba.v14i1.1285

**Published:** 2022-11-01

**Authors:** Ruth H. Leeney, Herinjaka Raveloson, Paul Antion, Vik Mohan

**Affiliations:** 1 Private, Rogil, Portugal; 2 Blue Ventures, Antananarivo, Madagascar; 3 Blue Ventures, Bristol, United Kingdom

**Keywords:** pandemic, holistic approach, livelihoods, symptom tracking, partnerships, resilience, adaptive capacity, rural community

## Abstract

Blue Ventures (BV) works holistically with communities in Madagascar, developing transformative approaches to catalyse and sustain locally led marine conservation. In response to the coronavirus disease 2019 (COVID-19) pandemic, BV’s priority was to safeguard the immediate wellbeing and livelihoods of as many communities as possible, recognising that livelihoods are integral to broader well-being. This article describes in detail BV’s health response and the perceptions of BV’s Madagascar team regarding the successes and challenges of this effort. As a result of the combined efforts of BV teams across Madagascar and in the United Kingdom, the existing healthcare services at BV’s sites were maintained, and messages about recognising and dealing with COVID-19 and the importance of vaccination were conveyed to communities that might otherwise not have received comprehensive information. Data were also collected on suspected cases in areas where testing was not available, and outbreaks of suspected COVID-19 cases were managed. Because BV’s teams are embedded within the communities where they work, they maintain strong relationships with communities and conveyed important messages around reducing the spread of COVID-19, not only via activities in response to the pandemic but also through activities for other programmes such as fisheries and livelihoods. Blue Ventures’ holistic approach ensured that the organisation had a multidimensional understanding of the impacts of the pandemic on communities, facilitating the development of more relevant messaging that considered both safety and the need for continued income-generating activities. Staff felt that an effective public health response was facilitated by strong in-country partnerships and BV’s long-standing presence in communities.

**Contribution:**

The challenges in responding to the pandemic and in implementing and maintaining effective behaviour change are discussed. Although not an objective study of the effectiveness of the response or a comparison with other approaches, the lessons learned from this process are shared in the hope that they may inform responses to future shocks in low-income countries.

## Introduction

**FIGURE 1:**
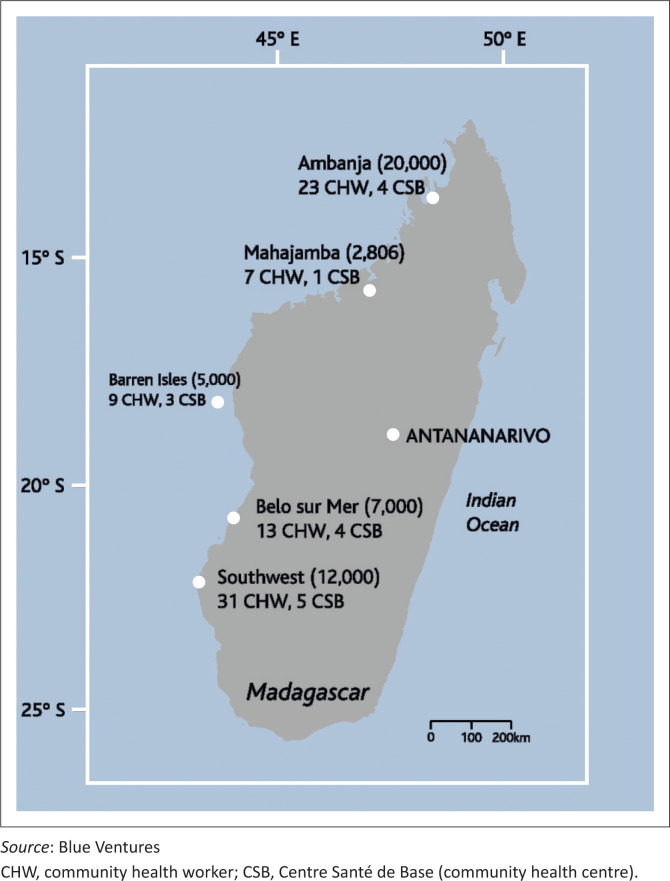
The zones where Blue Ventures operates in Madagascar and the number of community members (in parentheses), community health workers and community health centres supported at each site.

Blue Ventures (BV) is a human rights-based conservation organisation, working holistically with traditional fishing communities to restore the world’s oceans and improve communities’ livelihoods. The organisation currently serves 46 806 individuals in communities across five zones in Madagascar (Figure 1). Blue Ventures’ approach includes supporting locally led management of fisheries and marine ecosystems, supporting the development of alternative livelihoods (e.g. Robinson & Pascal 2009:38–42) and providing community-based health services. To address unmet health needs in Madagascar’s remote coastal communities, local women have been trained as community health workers (CHWs) to provide healthcare and education to their communities, with a focus on family planning, maternal and child healthcare, and safe water initiatives. Blue Ventures currently supports 83 CHWs and 17 community health centres (CSBs) in Madagascar (Figure 1). This multi-sectoral approach has led to increased access to family planning and uptake of modern contraceptives in previously underserved communities (Robson et al. 2017:77–78), to the continued provision of healthcare in the aftermath of a devastating cyclone (Maat et al. 2021:8–9) and to improvements in income from fishing (Oliver et al. 2015:11–13).

On 21 March 2020, the Malagasy government declared a public health state of emergency to address the coronavirus disease 2019 (COVID-19) pandemic. In the early stages of this period, it was unclear how COVID-19 would affect communities in Madagascar or in other low-income countries. Several months later, information compiled by BV and an understanding of the situation based on ongoing observations by in-country staff indicated that government assistance and household-level coping strategies were not sufficient to sustain pre-crisis living standards in Madagascar. Rural and urban areas in Madagascar are strongly linked through a bidirectional flow of goods such as food, labour and cash, which was interrupted by the measures introduced in urban areas to restrict movements (Blue Ventures, unpubl. data). This impacted the productive capacity and the livelihoods of people in peri-urban and rural areas and resulted in widespread food insecurity and challenging economic conditions for many (Egger et al. 2021:1–12). Small-scale fishers in Madagascar are already disproportionately impacted by a suite of interconnected challenges including poverty, destructive industrial fisheries and climate breakdown. Many of these communities are isolated from basic services, and COVID-19 threatened to be an additional test of their ability to adapt.

Pandemics can affect the economic, social and political stability of communities, nations and transnational organisations and thus, any response to a pandemic must minimise these effects, as well as taking every action to minimise mortality and morbidity (Rubin 2011:4). In addition to vastly different contexts in terms of socio-economics and state capability to support citizens, low-income and lower middle-income countries differ from high-income and higher middle-income countries not only in the availability of resources but also in having substantially younger age distributions (Alon et al. 2020:3–4; Hodgins & Saad 2020:137). The likely benefits, harms and feasibility associated with possible responses to COVID-19 are to some extent determined by differences in resources, age structure, stability and state capability. In the context of a low-income country such as Madagascar, a feasible, contextually appropriate strategy had to be developed to minimise any social and economic harm that might arise from health protection measures.

This article describes BV’s experience as a conservation organisation in developing a health response to COVID-19 in Madagascar and reflects on how the organisation’s existing cross-sectoral and collaborative approach facilitated this response. The article does not represent an objective assessment of this approach or a comparison of the perceptions of a range of stakeholders of its effectiveness. Rather, it presents the experiences of BV staff members in Madagascar (and several in the UK) who were involved in implementing and assessing this response on the ground. Their perceptions of what worked well and what was challenging, as well as how existing relationships with and infrastructure within communities may have facilitated the implementation of the organisational response, provide a valuable insight into the lived experiences of individuals balancing conservation and healthcare priorities during an unprecedented global pandemic. The lessons learnt and how the organisation has changed as a result are highlighted. While the authors acknowledge that it would have been desirable to assess and present the perceptions of community members involved in BV’s programmes alongside those of staff members, this was not possible given constraints on time and resources. It is hoped nonetheless that documenting the experiences of BV teams in Madagascar may serve to inform other NGOs in low-income countries, either as they continue to respond to COVID-19 or when responding to crises in the future.

## Methods

Semistructured interviews were used to gather information about the activities put in place by BV teams and their partners at the study settings (the sites in which BV runs programmes in Madagascar). Staff who were involved at various levels in the planning and execution of BV’s response to COVID-19, both in Madagascar and BV’s UK office, were interviewed over video call by one of the authors (R.H.L.). The interviews were based on a set of predetermined questions developed through consultation with all staff involved in the COVID-19 response. Interviewees were also encouraged to share their experiences of COVID-19 and BV’s response to the pandemic more broadly, in response to several open-ended questions. Interviews with 11 staff members (of whom eight were based in Madagascar or had been, prior to the pandemic) were conducted between 04 and 19 March 2021 and on 22 June 2021. Most or all of the COVID-19-related activities were ongoing at sites throughout Madagascar at the time of the interviews, and thus, data could be collected whilst the activities, their challenges, successes and impacts were still foremost in the minds of staff members. In early 2021, COVID-19 cases began to noticeably rise in Madagascar, and thus, the final interview in June 2021 was conducted to generate an updated understanding of the situation. These interview data were combined with information in routine programme reports, internal communications and data collected by programme staff on the progression of the pandemic.

### Ethical considerations

This article followed all ethical standards for research without direct contact with human or animal subjects.

## Results

### Planning and strategy development

In the early stages of the pandemic, guidance on appropriate response approaches was based largely on experiences in China and Europe and had not yet been adapted for resource-poor contexts such as subsistence farming and fishing communities, which have little social protection and where remote working is largely not applicable (Hodgins & Saad 2020). It was thus necessary to develop contextually appropriate, relevant guidance for fishing communities in Madagascar. This led to the encouragement of greater awareness, across all of BV’s sites, of what was happening with respect to the pandemic. Initially, the UK team collated as much information as possible on COVID-19, including the disease profile in the Global South and any advice on how to respond in a low-income country context. The connection that the BV health team had with existing global health networks proved invaluable in this respect. The Community Health Impact Coalition – a coalition of health organisations in over 40 countries which supports professionalised CHWs and access to quality healthcare – set up weekly meetings to share resources, information and experiences. This became an important source of information on other organisations’ experiences of and responses to COVID-19.

A database of reliable public health information was assembled, drawing on sources such as *The Lancet*’s hub of COVID information (and those of other scientific journals), the World Health Organization (WHO), Public Health England and the *Our World in Data* website. There was considerably less testing for COVID-19 in Madagascar and other low-income countries compared with higher-income countries (Hodgins & Saad 2020:138), meaning that COVID-19 cases were likely being under-reported (Schoenhals et al. 2021:1). Given the dearth of data, observations made by BV staff working at the community level were combined with official data on COVID-19 cases to build a more complete and continuously updated picture of the extent and impacts of the pandemic. Because of the importance of fisheries to the coastal communities of Madagascar, information from global fisheries organisations on potential impacts on fisheries and value chains was integrated into planning. So too was guidance issued by the Food and Agriculture Organization of the United Nations (FAO) and the responses of other organisations to that guidance.

These research and planning activities led to the establishment of three core objectives for BV’s response to the pandemic in Madagascar: to reduce transmission of COVID-19, to protect the most vulnerable and to strengthen health systems so that they are better able to withstand the pressure generated by the pandemic. These objectives were determined to be both realistic and critical to minimising mortality. In particular, the strengthening of local healthcare systems was seen to be important because of the known risk of higher rates of mortality from other causes that can occur as an indirect result of epidemics or other stressors on health care systems. For example, people were discouraged from seeking care in hospitals for anything other than COVID-19 in some low- and lower middle-income countries (Hodgins & Saad 2020:139–140), and research estimated that COVID-related disruptions to tuberculosis and HIV services could result in a 10% increase in HIV-related mortality and a 20% increase in tuberculosis deaths, because of interruptions in antiretroviral treatment and disruption of timely diagnosis and treatment, respectively (Hogan et al. 2020:e1132–e1141).

Blue Ventures has been developing livelihood alternative programmes in rural communities in Madagascar since 2007. In addition to the health-related response activities, the organisation recognised the importance of ensuring that livelihood programmes could continue to operate in a safe way, in order to ensure that key sources of income for remote communities were disrupted as little as possible. A framework to facilitate risk assessments for livelihoods was developed, incorporating thoughts on the likely biggest impacts of the pandemic on communities. Health and livelihoods teams worked in parallel to assess which established conservation and livelihood programmes could feasibly continue to operate, given the restrictions being implemented by the Malagasy government, and how to ensure participants’ safety.

Once the required new activities had been determined for each site, work plans were developed to incorporate them. In collaboration with Madagascar’s Population Health Environment (PHE) Network, resources (available at: https://partners.blueventures.org/covid-19-resources/) were developed on how to formulate cross-sectoral responses, assess community needs and develop public health messaging that communities could act on. This involved the development of guidance documents, tools and outreach materials. The coordination group in the UK developed and implemented the health response in coordination with the team in Madagascar. Health and livelihoods teams worked alongside the fundraising team to assess which established conservation and livelihood programmes could feasibly continue to operate, given the restrictions being implemented by the Malagasy government and the requirement that any ongoing activities be safe for participants. Good relationships with supportive donors meant that many were willing to remove restrictions on the use of their funds, allowing BV to divert resources to areas of greatest need.

### Health response activities implemented in Madagascar

To implement the response to COVID-19, BV worked in close collaboration with community leaders, the Ministry of Public Health (MPH) and several other actors, including BV’s partner NGOs, the PHE network, local vigilance committees, CHWs and members of each community. It proved important to involve and coordinate with local authorities, such as local mayors and heads of government health clinics (CSBs), in the activities being supported by BV. This was achieved through regular coordination meetings with stakeholders, monthly reporting to government health bodies and frequent *ad hoc* communication of important updates to stakeholders. Communications between BV, other organisations and the communities appeared to be more effective because of the presence of these local authorities. The interviewees believed that this was because of the authority and trust conferred to BV as a result of this endorsement. Table 1 provides a complete list of organisations and institutions with which BV collaborated to realise its COVID-19 response programme in Madagascar and the roles each organisation played.

**TABLE 1:** Collaborators and partners for Blue Venture’s COVID-19 response in Madagascar and their roles.

Partner name	Role
Madagascar Ministry of Public Health	Responsible for coordinating and overseeing the Government of Madagascar’s health response: pandemic surveillance; dissemination of information to health staff; management of supply chains; vaccination
PHE network	Sharing of information and resources from government agencies and between NGOs
Marie Stopes Madagascar (NGO partner)	Providing sexual and reproductive health services to partner communities
Community health centre staff	Providing vaccinations in collaboration with the USAID ACCESS programme
USAID’s ACCESS^1^ (Accessible Continuum of Care and Essential Services Sustained) program	Directly supported MPH in the implementation of vaccination points throughout Madagascar
PSI Madagascar	Responsible for the supply of medications, chlorine for water purification, and contraceptives used by CHWs
Vigilance committees, local government, and Mayor	Leading community sensitisation and support activities; authorising BV’s outreach work and response
Community health workers	Data collection; sensitisation, providing treatment; reporting COVID suspected cases to BV
UNICEF Maintirano	Distribution of handwashing materials and soap in the Melaky region
Morombe Archaeological Project (MAP)	Raised funds and supported response to April 2021 COVID-19 outbreak in Southwest Madagascar through the distribution of soap, face masks and community outreach
Ocean Farmers and Indian Ocean Trepang (IOT)	Supported the continuation of commercial alternative livelihood activities in the southwest by continuing to visit, provide technical support, and buy seaweed and sea cucumbers from community farmers
GIZ (international NGO)	Food distribution for vulnerable people in Ambanja Bay
Local resource management associations, youth groups, and women’s association	Raising awareness in their community; manufacturing facemasks and handwashing stations

BV, Blue Ventures; CHWs, community health workers; PHE, Population Health Environment; UNICEF, United Nations Children’s Fund; GIZ, Deutsche Gesellschaft für Internationale Zusammenarbeit; USAID, United States Agency for International Development; ACCESS, Accessible Continuum of Care and Essential Services Sustained programme.

#### Key actions implemented at community level

Table 2 summarises the COVID response activities implemented by BV, the communities with whom BV works and partner organisations at BV sites in 2020. The activities implemented by BV are presented here according to the three objectives of the organisation’s strategy.

**TABLE 2:** Activities implemented in response to COVID-19 at Blue Ventures sites in Madagascar (in 2020).

Response activity	Number
Face masks manufactured	6341
Posters distributed	2902
Cartons of soap distributed	192
Handwashing stations established	210 (in 100 communities)
Community health workers, vigilance committee members and redeployed BV staff trained in government policy on gatherings, social distancing	212
Door-to-door household visits by CHWs, vigilance committee members and redeployed BV staff to disseminate public health information, ensure soap and water is available for handwashing, reinforce preventive practices, etc.	2665
Community health workers trained in data collection on COVID-19, collecting data on suspected cases and reporting to BV	84
Community health workers and VC members trained in the ‘no-touch’ protocol	105
CHWs trained in administering COVID-19 vaccine	83
Health centres trained in providing treatment for COVID-19	17
Existing health centres supported to provide vaccination	17
CHWs and vigilance committees reporting COVID-19 and RMA data to BV	101

CHW, community health workers; BV, Blue Ventures; VC, vigilance committee; RMA, regional management authority.

**Reducing community transmission:** At the community level, BV communicated guidance for minimising the risk of infection and spread of COVID-19 to communities via a diverse array of communication channels, including posters, songs and music videos, public broadcasting of the president’s fortnightly speeches regarding the national State of Emergency, radio slots and home visits by CHWs and members of the vigilance committees. The key points in this guidance were given as follows:^1^

- *Social distancing*: People were encouraged to keep a 1 m distance from people outside of their household.
- *Masks*: People were encouraged to wear masks at community meetings, when going to the market and in any situation where there were gatherings of people.
- *Handwashing*: Washing stations were provided to all of the communities in which BV works, and community members were advised to regularly wash their hands.
- *Limitations on gatherings*: Initially (from March until September 2020), all public gatherings were banned. As restrictions eased, BV sets limitations on the number of people at a single gathering during activities for which BV was the organiser.
- *Symptoms*: People were encouraged to report symptoms via a free national hotline, but many people do not have access to a telephone in Madagascar. Anyone with symptoms was also advised to self-isolate at home and to contact their local CHW, who could provide advice and recommend treatment at health centres. The CHWs monitored the number of COVID-19 cases, the number of people with symptoms and what was happening to those individuals.

Community vigilance committees were set up in each community. Each committee comprised a group of between 10 and 20 individuals, led by the local CHW and including several senior community members. All community members with some sort of leadership responsibility or involvement with health services were invited to join these committees. Their role was to ensure ongoing community involvement in monitoring and detection of COVID-19 cases, to take early action to report suspected cases and respond to those cases. They ensured that households were aware of the restrictions designated by the government and were reminded of social distancing and other safety measures. They also reminded communities to report suspected cases to CHWs, who would then conduct clinical assessments (see Section ‘Communications’ for details).

**Shielding the vulnerable:** To support vulnerable individuals to stay at home, packs of essential household items (rice, oil, sugar and candles) were distributed in communities where BV works in northwest Madagascar, in partnership with the German NGO GIZ. Those who were eligible for this support included pregnant women, elderly individuals and those with pre-existing health conditions or respiratory conditions. In 2020, a total of 2709 individuals (345 pregnant women, 408 individuals with underlying health conditions or respiratory problems and 1956 elderly people) were encouraged and supported to stay at home and minimise social contact. Food, soap, social support and public health information about COVID-19 were provided to vulnerable individuals and their familiesin all five regions where BV works, ensuring that their basic needs were met whilst minimising their exposure to the coronavirus.

**Strengthening the health system:** A core part of BV’s response was to ensure that existing healthcare services could continue to be provided throughout the pandemic, with modifications where necessary. This maintenance of community health services, including antenatal and postnatal care and the provision of contraception, has been a key success of BV’s response. To facilitate women accessing contraception without visiting clinics, BV introduced the use of an injectable contraceptive that has been licensed for self-administration (medroxyprogesterone acetate), which was developed for resource-poor settings where access to health services is limited. It was already in use in Madagascar pre-pandemic, but self-administration had not been facilitated, so BV provided training on this to 83 CHWs in 80 villages. At the time of writing, at least 4270 women were using this method, and it had been approved by the MPH, which also began encouraging self-administration in other parts of the country. In addition, the supply chain of medication provided to CHWs and CSBs was maintained with support from local health clinics and the NGO Population Services International.

Given the lack of testing capacity throughout Madagascar and especially in rural areas, BV recognised that collecting data on COVID-19 cases via symptom tracking would be an important part of understanding the spread of the virus, and would inform how to respond appropriately at the regional level. Blue Ventures therefore engaged with the MPH to support and facilitate training for CHWs in the surveillance and monitoring of COVID-19. Training was provided to CHWs by the District Health Services (themselves trained by the Department of Health Monitoring, Epidemiological Surveillance and Response [DVSSER] from the MPH). It covered basic information about what COVID-19 is; sanitation, tracking and collecting data on symptoms of the virus; and how to mobilise the community to be vigilant about the virus and to share information on possible cases with BV. These data allowed BV to track suspected cases (people with symptoms) at each site, collect data on the number of vulnerable people (people above the age of 50, pregnant women, other people with respiratory diseases like TB) and record general mortality rates. The health surveillance activities (*veilles sanitaires*) overseen by CHWs included the following activities:

- early identification of disease cases and unusual events
- notification to the nearest health facility
- investigation of COVID-19 cases or outbreaks and contact tracing
- facilitating communication for the adoption of health-promoting behaviours
- collecting data on suspected COVID-19 cases and reporting those data to BV and the Ministry of Health
- updating data on suspected cases by region or community and classifying regions according to those data
- encouraging symptomatic people who were unwilling to self-isolate to follow public health guidance.

Data collection was conducted by 83 CHWs and health centres, as well as by 17 vigilance committee members (Figure 1), and as of July 2021, it continues at all sites. The data provided to BV by CHWs on numbers of suspected cases were shared with the MPH to support government efforts to track and manage the pandemic. They were also used by the organisation to periodically assess the situation in each area, modify guidance and provide additional support when needed.

To reduce client-to-clinician transmission of the virus, BV supported the development of a ‘no-touch protocol’ – a clinical protocol designed to keep 2 m distance between health workers and clients. Community health workers had no way of protecting themselves when attending to clients before the arrival of PPE, so this protocol was seen as an important measure in reducing virus transmission. However, the use of this protocol was not widely adopted (see section ‘Challenges’). Witnessing this limited adoption, BV supported the development of a compromise approach, the ‘low-touch’ protocol, an approach that serves to minimise client–clinician contact.

#### Communications

The majority of communities that BV works with in Madagascar are located in remote parts of the country. In some communities, there may be no access to television, and not all community members have access to radio, so international and even national news does not always reach these areas or may be significantly delayed. Blue Ventures teams thus recognised the importance of ensuring that all official, government-issued announcements regarding the pandemic were shared with all community members. For example, in the south-west, the president’s speeches regarding the national State of Emergency (which were issued fortnightly to update restrictions and advice) were recorded by the BV team and broadcasted over a sound system in each village they visited. This helped to assure communities that BV’s messaging about the pandemic was in line with that issued by national authorities.

Limited internet connectivity on much of Madagascar’s west coast meant that the use of social media messaging to increase the adoption of preventive measures during the pandemic was not viable within the communities BV works with. Posters conveying information about the transmission of COVID-19 and recommended precautions were produced in the local dialects of each region. The key messages of these posters were also represented graphically, alongside the text, to ensure that these messages reached individuals of all literacy levels. Music, especially songs in Malagasy and local dialects, has proven an effective way to transmit conservation messages in the past, so locally composed songs (shared via CD and USB drives with community members) and music videos to explain the virus, the safety measures people should take and (in 2021) vaccination were developed and proved popular. Slots on local radio stations allowed doctors, health workers and local authorities to speak about their experiences with the pandemic and to assure people who had not directly experienced the pandemic in their own communities that it was real. This proved important as many rural communities did not experience high levels of COVID-19 infections in 2020, and after several months, disbelief around the existence of the illness began to grow.

#### Evolution of the response

At the time of the initial consultation for this study, the pandemic had not had the expected impact in Madagascar, meaning that BV had raised awareness and prepared communities for a situation that had, at that point, few health impacts (and only a short-lived impact on livelihoods) on those communities. Blue Venture’s approach thus had to incorporate ongoing vigilance as well as adaptation and responsiveness to new situations. In February 2021, the second wave of COVID-19 reached Madagascar. Testing for COVID-19 was largely unavailable in rural areas, so infection rates were not quantifiable, but medics in CSBs reported seeing more ill people than in 2020 and recognised the symptoms of COVID-19 in many of these people. Blue Ventures staff observed that the frequency with which face masks were being worn in rural communities had decreased significantly, whilst other measures that had been introduced – such as hand-washing – were still largely being practised. In response to this, sensitisation efforts were redoubled in the communities where BV works, with efforts focusing on encouraging continued mask use.

In April 2021, the MPH approved its National Vaccination and Deployment Plan for COVID-19. Beginning in June 2021, vaccines were made available to communities throughout Madagascar. To further support the Ministry’s efforts to address COVID-19, BV staff have undertaken several activities related to vaccination. Consultations have been carried out with both the vaccination department of the MPH and with USAID’s ACCESS programme. Blue Ventures has conducted community meetings to provide accurate information to communities about the vaccine, explain its role in combatting COVID-19 and inform people of the vaccination centre locations. Blue Ventures has also facilitated the transport of official vaccination staff to the remote communities where BV works. As of August 2021, 2131 community members and 94 BV staff members had received the first dose of the two-dose Covishield vaccine at BV sites across Madagascar.

### Reflections on response effectiveness – successes

Blue Ventures works by supporting communities, developing strong teams consisting largely of local staff members and building partnerships with local organisations. These approaches characterised the development and delivery of BV’s response to COVID-19 and may have made these response activities more effective, although without a point of comparison it is not possible to qualify the relative efficacy. Having teams on the ground and based permanently at the sites where they worked meant that BV’s staff were able to keep working during the early stages of the pandemic, when for safety reasons, other international organisations had to withdraw or even evacuate staff. This meant that BV could continue many of their longer-term programmes, albeit in modified or limited ways, as well as implementing COVID-19 response activities. Strong in-country partnerships with NGO partners and local governments greatly facilitated the response activities.

The holistic nature of BV’s work in Madagascar facilitated the ambitious and multidimensional response that BV effected. Because the organisation works not only in the health sector but also on livelihoods, fisheries management and conservation, staff from other programmes could be rapidly redeployed to assist in the health response, which effectively tripled the number of staff available to contribute to the COVID-19 response efforts. The knowledge of community livelihoods, behaviours and practices that the organisation has as a result of its fisheries, livelihoods and conservation programmes also facilitated the crafting of appropriate messaging for these communities. For example, knowing the importance of fishing as the primary livelihood for the coastal communities BV works with, and knowing how often and with whom people go fishing and market their catches, BV staff were able to develop specific guidance for fishers that would minimise the risk of spreading the virus, without stopping them from fishing. This meant advising people only to go fishing with other members of their household and only one individual per household going to market to sell fish, whilst observing social distancing and mask-wearing protocols. In addition, the continuation of activities such as the community meetings for livelihood programmes (when safe to do so) meant that there were additional fora in which BV teams could deliver advice and updates relating to COVID-19. The large numbers of people reached with safety messaging and the level of behaviour change effected is unlikely to have been possible without BV’s multisectoral approach, which engages a large array of stakeholders and broad community representation. During the period of greatest compliance with COVID-19 protocols, an estimated 40% of all community members were wearing masks when they went fishing or to market, and a large proportion of people were following handwashing protocols (measured through direct observation, by BV staff and vigilance committee members, of fishing and market activities).

Many staff felt that BV’s existing holistic approach proved helpful in dealing with the multiple and diverse impacts that the pandemic posed. Blue Ventures’ site leaders and regional managers have an excellent understanding of how the organisation’s diverse programmes and activities intersect. This structure allowed for site-specific understanding of the impact of the pandemic and of interactions between impacts on health and livelihood and site-specific judgements regarding necessary precautions and the feasibility of activities. Because the impacts were not only on people’s health but also on the livelihoods of people already living in poverty, it was essential to not only address health issues but also maintain livelihood programmes where possible, introducing alternative income-generating opportunities where necessary. Staff who were redeployed from aquaculture and livelihood programmes to support the health teams brought their knowledge of the livelihood issues communities were facing to the health response, strengthening this cross-sectoral understanding.

Blue Venture’s existing community health staff, all community health practitioners with experience of working in public health and/or community health, were well placed to engage with government systems, partners and communities on matters relating to the pandemic. The existence of a health programme pre-pandemic meant that there was already capacity for healthcare at each site, and BV’s existing partnerships with in-country health organisations further supported the organisation’s response. All of BV’s CHWs were trained in data collection on COVID-19, symptomatic diagnosis and the no-touch protocol. The no-touch protocols have been approved by the MPH and are being adopted nationally. Blue Ventures also became the first NGO in Madagascar to facilitate, via partnerships with health organisations, the self-administration of injectable contraception.

### Reflections on response effectiveness – challenges

Several BV staff members encountered disbelief in the existence or severity of COVID-19 in their communities and found it difficult to change people’s attitudes. Many community members did not feel they needed to prevent the spread of the virus, perhaps because of the scarcity of cases in some communities in 2020. Staff thus found it increasingly difficult to encourage people to observe infection control practices. In early 2021, in response to the increasing number of COVID-19 cases and suspected cases throughout Madagascar, BV teams repeated the sensitisation work they had undertaken in 2020, to ensure that communities were once again reminded of the safety guidelines.

Finally, although the no-touch protocol developed by BV worked well at the community level, the low numbers of COVID-19 cases in rural communities meant that it was felt unnecessary to adopt the protocol. Instead, a ‘low-touch’ approach, which minimises but does not fully eliminate client–clinician contact, has been encouraged and is currently being used.

## Discussion

An analysis of the response to the 2018–2020 Ebola crisis in DRC suggested that the knowledge of local frontline health workers should have been incorporated and acted upon (Mayhew et al. 2021:1743). Experiences in Madagascar and several other African countries have indicated that a community-based system of healthcare is more effective in the prevention, early diagnosis and primary care in response to zoonotic and infectious diseases associated with extreme weather events (Maat et al. 2021:1–16). Similarly, BV’s response to the COVID-19 pandemic was heavily dependent upon local expertise and the involvement of trusted local actors. Mayhew et al. (2021:1738) also highlighted the importance of principles of reciprocity and respectful relationships as well as relatable messaging, principles that were at the core of BV’s response to the COVID-19 pandemic. This response is ongoing and will evolve in parallel with the situation in Madagascar. Nonetheless, BV’s experience thus far in developing this response has highlighted some valuable lessons around the links between organisational structure and scope and the ability to respond to a shock such as the pandemic.

The pandemic brought about a more purposeful engagement by BV with the existing global health network, which will prove invaluable for accessing relevant resources and support in the future. It also confirmed the value of including health programmes as part of a cross-sectoral approach to marine conservation. Social capital was built amongst communities in Madagascar, as BV teams worked to support communities to deal with the pandemic. Through activities such as ensuring that community education and sensitisation were done in a contextually appropriate way, supporting local capacity-building to respond to the pandemic, supporting disease surveillance and working to reduce client–clinician spread of the virus, BV became a highly visible actor in the public health space during the pandemic. This was formally recognised by the MPH and has served to strengthen the organisation’s relationship with the Ministry. In addition, the support around responding to COVID-19 that BV gave to fisheries organisations outside of Madagascar has led to potential new partnerships, as organisations see the value of integrated health and fisheries initiatives.

Maintaining the basic health services that communities used, including family planning services, prior to the pandemic, was essential to minimising the indirect impacts of COVID-19 on broader community health. Weinberger et al. (2020:169) noted the potentially devastating outcomes of failing to meet women’s needs for contraception during the pandemic. Not only were contraception services maintained but also the method of self-administration was introduced in response to the pandemic, and this will provide women with greater choice and independence in terms of their reproductive health. The adoption of this method by the MPH suggests that it is likely to also benefit communities beyond BV’s reach.

Careful efforts to maintain the operations of alternative livelihood programmes in a safe way, as well as strong relationships with commercial partners who were willing to continue operations and were flexible to meet BV’s response requirements, meant that livelihood programmes could continue even when government restrictions were most severe, and people’s incomes were minimally interrupted. Encouraging households to save within the structure of savings and loans groups will also help to build resilience to shocks, at least in the short term.

The need to respond to COVID highlighted the importance of disaster risk planning. The west coast of Madagascar is frequently subject to cyclones, and in recognition of the need to be better prepared for future shocks, a protocol is being developed to track events such as cyclones and to anticipate and mitigate impacts on programmes such as sea cucumber farming. Some of the infrastructure established as part of the COVID-19 response, such as the vigilance committees, will also help in coordinating responses to climate-related shocks and stressors. Staff in Madagascar noted that the pandemic has meant that they have gained experience in dealing with a crisis, and there will be an ‘organisational memory’ of this experience. Blue Ventures has also recognised the need for relationships with the relief sector and a knowledge of the sector’s activities and development in the future. The organisation is well placed to connect the communities it works alongside with relief organisations, should the need arise.

Community resilience can be defined as the collective ability of a neighbourhood or geographically defined area to deal with stressors and efficiently resume the rhythms of daily life through cooperation following shocks (Aldrich 2012). Such resilience is essential in low-income countries where external support may be limited, or where access by aid agencies may be challenging. Whilst some studies have suggested that communities reliant on NGOs or government can lead to reduced resilience to shocks, an effective collaboration between communities and NGOs, leading to long-term capacity building and sustainability, may make communities more resilient (Walters et al. 2021:69).

The multi-sectoral approach taken by BV, which recognises the interconnections between human populations, their health and their environment, has led to improvements in health. These improvements are likely to have contributed to community resilience, as communities composed of mostly healthy individuals are more resilient, allowing less healthy individuals to rely on healthier individuals for physical and emotional support (US Department of Health and Human Services 2009:5–6). In contributing to this study, several BV staff members noted that the pandemic demonstrated to some communities their own resilience and their abilities to adapt their way of life. BV’s approach of building capacity and skills within communities may also have contributed to the effectiveness of the response to COVID-19.

Whilst clear benefits of multisectoral working have emerged through this work, it is possible that a well-resourced public health organisation could have provided a better community health response to the pandemic. An approach that combines the technical expertise and organisational capacity of a public health specialist organisation with a holistic, community-led response may provide the optimal conditions for an effective pandemic response.

## Conclusion

The pandemic has brought about a far greater understanding and recognition of the link between human health and environmental health. It has also revealed the weaknesses in public health systems around the world. These lessons have reached all sectors of society and may have significantly changed how NGOs working in the health and environment fields operate. Shocks such as pandemics and the impacts of climate breakdown are only likely to increase in frequency and severity in the future (e.g. Bathiany et al. 2018:1–10). The experience of BV during the COVID-19 pandemic suggests that holistic programmes that support communities, not simply through health services or livelihood projects but via a multifaceted approach, may make organisations and communities more capable of responding to future shocks.

The pandemic has also highlighted the importance of self-determination and recognition of the rights of local communities, which allow them to meet subsistence needs during lockdowns and to help community members and neighbours to sustain livelihoods and nutritional requirements (Walters et al. 2021:67–69). The recognition of community rights and building of intracommunity skill sets for resource management, communication, health care and innovation linked to livelihood diversification will be critical in ensuring that communities are resilient to future shocks. Mainstream approaches to aid and development have been largely top-down and oriented towards narrowly defined economic goals, but the pandemic has revealed the importance of inclusive and egalitarian approaches and the need for development approaches that anticipate and respond to future, uncertain shocks, including pandemics, climate-related disasters, financial turbulence or something as yet not experienced (Leach et al. 2020:9–10). The holistic approach taken by BV, which views community needs and rights as integral to addressing conservation issues, is a model that may benefit other organisations working in contexts where future shocks may otherwise seriously compromise their ability to work effectively and threaten community well-being and survival.

## Footnotes

1 ACCESS is a 5-year programme that aims to improve and expand health services for the Malagasy people. Madagascar’s COVID-19 vaccination programme is managed solely by the Ministry of Health and ACCESS.
